# Bilateral Foveal Cysts in Mucopolysaccharidosis Type I (Hurler Syndrome): Response to Acetazolamide With Insights From Multimodal Retinal Imaging and Electrophysiology

**DOI:** 10.1155/crop/5349202

**Published:** 2025-11-19

**Authors:** Rashmi Lobo, Ahmed Al-Hinai, Aisha Al Busaidi

**Affiliations:** Department of Ophthalmology, Sultan Qaboos University Hospital, University Medical City, Muscat, Oman

**Keywords:** acetazolamide, bull's eye maculopathy, foveal cyst, mucopolysaccharidosis Type 1, multimodal retinal imaging

## Abstract

**Objective:**

The aim of this study is to report a case of bilateral foveal cysts in MPS I-H resolving with oral acetazolamide and to highlight the diagnostic value of multimodal retinal imaging and electrophysiological testing.

**Introduction:**

Hurler syndrome (mucopolysaccharidosis Type I-H) is a lysosomal storage disorder that can cause progressive multisystem complications. Retinal involvement often mimics retinitis pigmentosa (RP), and pathology may progress even after early hematopoietic stem cell transplantation (HSCT) due to limited enzyme penetration into ocular tissues.

**Case Summary:**

A 17-year-old female with MPS I-H, post-HSCT at 21 months, presented with bilateral visual decline despite a normal clinical fundus exam. Evaluation included spectral-domain OCT (SD-OCT), fundus autofluorescence (FAF), multifocal electroretinography (mfERG), full-field ERG (ffERG), and visual evoked potential. SD-OCT revealed bilateral intraretinal foveal cysts without leakage on fluorescein angiography. FAF showed a bull's eye maculopathy pattern; mfERG showed bilateral macular dysfunction. ffERG revealed rod–cone dystrophy. Two 3-month courses of oral acetazolamide (125 mg three times daily) led to complete cyst resolution and visual improvement.

**Conclusion:**

This case supports the role of systemic carbonic anhydrase inhibitors in treating nonleaking macular cysts in MPS I, similar to RP-related cystoid macular pathologies, and highlights the value of integrating electrophysiological and multimodal imaging, especially in occult retinal disease.

## 1. Introduction

Mucopolysaccharidosis Type I (MPS I) is an autosomal recessive lysosomal storage disorder caused by *α*-L-iduronidase deficiency, leading to systemic accumulation of glycosaminoglycans (GAGs), including in ocular structures [1]. Hurler syndrome, the most severe phenotype, typically presents with corneal clouding, glaucoma, optic nerve abnormalities, and progressive retinal degeneration [1, 2]. While early hematopoietic stem cell transplantation (HSCT) can improve systemic and neurological outcomes, its effect on ocular complications is limited due to restricted enzyme penetration into ocular tissues [3].

Retinal involvement in MPS I often mimics retinitis pigmentosa (RP), with rod–cone dysfunction on electroretinography (ERG) and structural changes such as retinal pigment epithelium (RPE) mottling, vascular attenuation, macular atrophy, and sometimes pigmentary changes. Electrophysiologic changes may precede or outweigh ophthalmoscopic findings, highlighting its value and that of multimodal imaging, namely, spectral-domain optical coherence tomography (SD-OCT) and fundus autofluorescence (FAF) for early detection [4].

High-resolution SD-OCT has increasingly revealed intraretinal fluid accumulation and cystoid macular edema-like lesions in patients with MPS [4, 5]. Despite such findings, the management of cysts in MPS is rarely addressed in the literature. Most reported cases have resolved spontaneously, and in one case, there has been partial improvement with topical NSAIDs [6, 7].

We present a case of bilateral foveal cysts in MPS I Hurler syndrome successfully treated with oral acetazolamide. The case highlights a potential therapeutic role for carbonic anhydrase inhibitors (CAIs) in managing macular cysts in MPS and underscores the importance of comprehensive structural and functional retinal evaluation.

## 2. Case Presentation

We report the case of a 17-year-old Omani female with Hurler syndrome (MPS I-H). Her diagnosis was confirmed at 15 months of age through a lysosomal enzyme assay, which revealed markedly reduced *α*-L-iduronidase activity (0.34 *μ*mol/g/h; normal range: 10–50 *μ*mol/g/h). Genetic testing identified a homozygous missense variant in the *IDUA* gene, c.590G>T (p.Gly197Val), currently classified as a variant of uncertain significance.

At 21 months of age, she underwent peripheral blood stem cell transplantation from an HLA-matched sibling. Following transplantation, she demonstrated significant systemic improvement, with sustained complete donor chimerism while off immunosuppressive therapy. She has never received enzyme replacement therapy (ERT).

Phenotypically, the patient displays features consistent with Hurler syndrome, including short stature, coarse facial features, gibbus deformity, and pectus anomalies. Systemic complications include adenoid hypertrophy with conductive hearing loss, mitral and aortic valve regurgitation with left ventricular hypertrophy, and psychosocial challenges related to her physical appearance.

Ophthalmic follow-up began at 10 months of age for bilateral mild corneal clouding. She was diagnosed with secondary open-angle glaucoma, with moderately elevated intraocular pressure (IOP) and increased cup-to-disc (C:D) ratios. IOP has since remained well controlled on fixed-combination latanoprost–timolol and brinzolamide eye drops. Fundus examinations were consistently unremarkable, and visual evoked potentials (VEPs) were normal at the age of 4 years.

At age 17, during a routine glaucoma follow-up, she reported decreased vision. Best corrected visual acuity (BCVA) had declined from 0.67 to 0.40 in both eyes. IOP was normal, and corneal clouding remained stable. Mild posterior subcapsular cataracts were noted bilaterally. Fundus examination showed stable discs with C:D ratios of 0.6 and no signs of pigmentary retinopathy or maculopathy.

**Figure 1:**
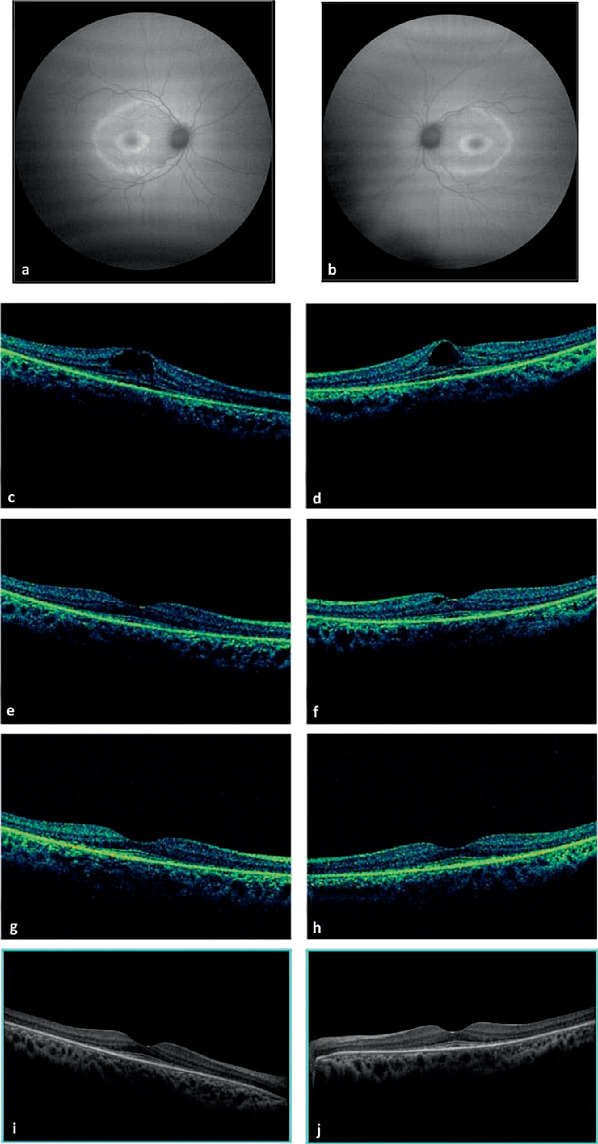
(a–j) Multimodal imaging in a 17-year-old patient with mucopolysaccharidosis I-Hurler syndrome treated with oral acetazolamide. Fundus autofluorescence (FAF) of the (a) right and (b) left eyes showing two concentric hyperautofluorescent rings consistent with bull's eye maculopathy. Spectral-domain optical coherence tomography (SD-OCT) at presentation of the (c) right and (d) left eyes revealing bilateral intraretinal foveal cysts. SD-OCT of the (e) right and (f) left eyes after 3 months of acetazolamide treatment, showing complete cyst resolution in the right eye and a residual cyst in the left. SD-OCT of the (g) right and (h) left eyes following two consecutive 3-month courses of acetazolamide, demonstrating complete bilateral cyst resolution. High-resolution SD-OCT of the (i) right and (j) left eyes showing perifoveal and parafoveal loss of the external limiting membrane (ELM) and inner segment/outer segment (IS/OS) junctions, thinning of the extrafoveal outer nuclear layer (ONL), thickening of the subfoveal ELM, and preservation of the subfoveal IS/OS junctions.

SD-OCT revealed bilateral intraretinal foveal cysts (Figure 1b). Fundus fluorescein angiography (FFA) showed no leakage. She was started on oral acetazolamide (125 mg three times daily) for 3 months while continuing her usual glaucoma medications. Five months later, the cyst in the right eye had resolved completely, but a small residual cyst remained in the left eye (Figure 1c). A second 3-month acetazolamide course led to complete bilateral cyst resolution (Figure 1d) and BCVA improvement to baseline of 0.67, likely limited by the underlying cataracts. Representative imaging findings are illustrated in Figure 1.

Additional SD-OCT findings included thinning of the extrafoveal outer nuclear layer (ONL), along with parafoveal and perifoveal disruption of the external limiting membrane (ELM) and inner segment/outer segment (IS/OS) junctions. In contrast, the subfoveal ELM appeared thickened, with preservation of the subfoveal IS/OS junction (Figure 1e). FAF revealed two concentric hyperautofluorescent rings at 1.7 and 3.7 mm from the foveal center, consistent with a bull's eye maculopathy pattern (Figure 1a).

Visual field (10–2) testing was unreliable. Flash VEP showed severely reduced P2 amplitude in the right eye and moderate reduction in the left. The implicit time of P2 was normal bilaterally. Full-field ERG showed moderate to severe rod–cone dysfunction bilaterally that was worse in the right than in the left. Multifocal ERG showed reduced paracentral responses in both eyes and additional central depression in the right eye.

At 6-month follow-up after stopping acetazolamide, the patient remained stable with no cyst recurrence, well-controlled IOP, and preserved visual acuity.

## 3. Discussion

We describe the first documented case of visually significant bilateral foveal cysts in Hurler syndrome (MPS I-H) resolving with systemic acetazolamide. Although intraretinal cystic changes have been increasingly reported in MPS using SD-OCT [4, 5], their pathophysiology and optimal management remain poorly defined [6], with most cases resolving spontaneously and only a single report showing partial improvement with topical nepafenac in Scheie syndrome (MPS I-S) [7]. Our case provides novel evidence that oral CAIs may be effective for MPS-related macular cysts.

The patient's retinal phenotype aligned with previous descriptions of MPS I retinopathy, which often mimics RP [4]. Despite clinically unremarkable fundi, electrophysiology revealed significant rod–cone dysfunction, highlighting the diagnostic importance of ERG and mfERG in detecting subclinical retinal disease [4, 8]. Multimodal imaging revealed a bull's eye maculopathy pattern with concentric hyperautofluorescent rings corresponding to zones of ellipsoid and ELM disruption, a phenotype now increasingly recognized in MPS I and previously described in RP [9, 10]. Thinning of the extrafoveal ONL was also observed in our case, consistent with the novel structural phenotype described by Magalhães et al. [1]. These structural observations were corroborated by mfERG, which demonstrated reduced paracentral responses in both eyes and greater central involvement in the right. These observations emphasize that structural appearance on fundoscopy may underestimate functional impairment, underscoring the value of combined structural and functional assessment.

A notable finding was thickening of the subfoveal ELM, thought to reflect Müller cell activation in response to GAG accumulation [6]. This feature may represent an early biomarker of MPS-related retinal change.

The choice of acetazolamide was guided by its established role in managing nonleaking cystoid macular changes in RP [11]. Its proposed mechanism—enhancing fluid resorption across the blood–retinal barrier—supports its use in noninflammatory, degenerative cysts [12]. Although topical CAIs such as dorzolamide may be considered an alternative first line [11], our patient developed cysts despite concurrent topical CAI, suggesting the need for a trial of systemic treatment. Interestingly, the eye with worse ERG dysfunction showed faster cyst resolution, echoing prior observations that photoreceptor degeneration severity does not directly predict cyst persistence [13].

An important consideration in this case is the potential role of prostaglandin analogs. Latanoprost has been associated with cystoid macular changes [14]. While this cannot be excluded as a contributing factor, the absence of angiographic leakage, the noninflammatory nature, and the complete response to acetazolamide while still on latanoprost support an MPS-related mechanism rather than drug-induced cystoid edema.

## 4. Conclusion

Oral acetazolamide may offer an effective therapeutic option for intraretinal macular cysts in MPS I. This case demonstrates complete anatomical and visual functional recovery, an outcome not previously reported. Our findings highlight the importance of integrating structural (SD-OCT and FAF) and functional (ffERG and mfERG) retinal assessments in MPS and suggest that treatment strategies used for RP-related CME may be applicable in MPS-related macular cysts.

## Data Availability

No new data were created or analyzed in this study. Data sharing is not applicable to this article.
